# Retinal pigment epithelium pseudopods reach across the divide to maintain photoreceptor performance and save vision

**DOI:** 10.1172/JCI205127

**Published:** 2026-04-01

**Authors:** Akrit Sodhi

For decades, the field of retinal cell biology has been governed by a fundamental proximity paradigm: the metabolic and structural integrity of the hyperspecialized photoreceptor cells is strictly dependent on close, nanometer-scale apposition with the retinal pigment epithelium (RPE), analogous to the relationship between a high-performance Formula 1 race car and its pit crew. This close interface is essential for the daily phagocytosis of shed photoreceptor outer segment (OS) discs, a process critical for preventing accumulation of photooxidative debris. It was long posited that any significant physical separation — whether through subretinal effusions or the accumulation of deposits — would inevitably lead to a failure of OS renewal, subsequent neurodegeneration, and vision loss (1, 2).

However, the proximity paradigm raises a clinical paradox: how do patients with vitelliform macular dystrophy or subretinal drusenoid deposits maintain high visual acuity despite a marked expansion of the subretinal space (3, 4)? In a paper published in this issue of the *JCI*, Lewis et al. (5) used the *Adam9*-deficient mouse, a model of retinal degenerative disease, to reveal an unexpected explanation to this long-standing question.

In the *Adam9*^−/−^ model, Lewis and colleagues demonstrated that the subretinal space expands early in development, yet the rate of OS renewal remains indistinguishable from that of WT mice (5). They further provided evidence attributing this to a specialized compensatory mechanism: the formation of elongated RPE pseudopods. By extending apical projections across the pathological void to physically engage and phagocytose photoreceptor OS, the RPE exhibited a previously unrecognized degree of morphological plasticity. The photoreceptors still capture the (spot)light in the parc fermé, to extend the Formula 1 analogy, but this discovery redefines the RPE as a highly dynamic cellular monolayer capable of active structural remodeling to preserve the renewal cycle, effectively shifting the biological focus from static proximity to functional connectivity.

This research further offers a critical reappraisal of the innate immune response within the degenerating retina. A prevailing hypothesis in contemporary neuroscience suggests that subretinal mononuclear phagocytes (MPs) serve a neuroprotective or “auxiliary support crew” role, potentially augmenting RPE phagocytosis during stress (6). But Lewis and colleagues provided direct evidence to the contrary. Despite a massive migration of MPs into the expanded subretinal space, these cells did not contribute to OS disc ingestion. Experimental depletion of MPs had no appreciable effect on photoreceptor survival or function, suggesting that in certain degenerative contexts, MPs may be spectators (or, at best, figurative Paddock Club guests) rather than active participants in retinal rescue, urging a more cautious interpretation of the immune system’s role in retinal homeostasis.

By identifying RPE pseudopod formation as the mechanism sustaining vision in the presence of subretinal expansion, this study provides a biological foundation for the slow progression of diseases like Best disease, age-related macular degeneration, and central serous chorioretinopathy. Understanding the molecular triggers of this RPE plasticity may unlock therapeutic avenues aimed at enhancing this “long-distance” renewal process, potentially extending the functional lifespan of photoreceptors in patients with blinding degenerative diseases.

## Version 1 04/01/2026

Electronic publication
